# Attitudes Toward HPV Vaccination in Sweden: A Survey Study

**DOI:** 10.3389/fpubh.2022.729497

**Published:** 2022-05-10

**Authors:** Maria Wemrell, Lena Gunnarsson

**Affiliations:** ^1^Department of Gender Studies, Lund University, Lund, Sweden; ^2^Unit for Social Epidemiology, Department of Clinical Sciences, Lund University, Malmö, Sweden; ^3^School of Humanities, Education and Social Sciences, Örebro University, Örebro, Sweden

**Keywords:** HPV vaccination, vaccine hesitancy, information, attitudes, survey, Sweden

## Abstract

**Background:**

While HPV vaccination uptake in Sweden is quite high, at around 80%, vaccine hesitancy remains an issue in countries throughout Europe. The latter can be related to a contemporary context of increased contestation of expert knowledge and of a large share of information on health-related issues including vaccination today being sought *via* the internet. Still, there is a paucity of recent research on attitudes toward the HPV vaccine in a larger sample of the population in Sweden. This survey study assesses such attitudes and any correlations between vaccine hesitancy and sociodemographic characteristics, trust in healthcare and other societal institutions, and evaluation of the reliability of different sources of information.

**Methods:**

The validated survey questionnaire was distributed to adult women in Sweden (*n* = 2,000), *via* a nationally representative web panel. The response rate was 37%. Aside from descriptive statistics, associations between vaccine hesitancy and sociodemographic and other variables were computed using logistic regressions and expressed as odds ratios (ORs) with 95% confidence intervals (95% CIs).

**Results:**

Our results show a positive attitude toward HPV vaccination overall. Still, some degree of HPV vaccine hesitancy was indicated by 33.8% of the respondents, and more pronounced hesitancy by 7.6%. Regarding vaccination in general, a very positive attitude was indicated by 55%. HPV vaccine hesitancy was associated with low education and low income and strongly associated with a lack of confidence in healthcare and other societal institutions. It was also correlated with a self-assessed lack of access to, and ability to assess the origin, quality and reliability of, information about the HPV vaccine.

**Conclusion:**

Efforts to provide transparent information about HPV vaccination should be combined with healthcare providers being open to discuss vaccine concerns with patients and avoiding practices that do not promote trust.

## Introduction

In order to prevent human papillomavirus (HPV) infections and thus cervical cancer (1), and in accordance with recommendations from the WHO (2), many countries across the world have implemented national HPV immunization programs (3). In Sweden, after the HPV vaccine Gardasil was approved in 2006, in 2010 a school-based, free-of-charge quadrivalent HPV vaccination program for girls was introduced. Since then, HPV vaccination has been tied to a substantial reduction of the population level risk of invasive cervical cancer and genital warts (4–6). In 2020, boys were also included in the Swedish vaccination program, in order to decrease HPV contagion as well as HPV-related cancers, such as oropharyngeal and anal cancer (7), which also afflict men (8).

HPV vaccination coverage varies between countries, and remains suboptimal or has fluctuated in some (9–12). In Sweden the HPV vaccination coverage is quite high, at around 80% (8). The national goal of 90% has not been reached, however. As in other countries (13–16), coverage has been found to vary between sociodemographic groups in Sweden (17, 18). Such disparities have decreased after the introduction of the school-based HPV vaccination program, but a lower coverage among girls whose parents have low income, low education and migration backgrounds has persisted (18, 19).

HPV vaccine uptake is affected by public attitudes (20), and vaccine hesitancy is an issue in countries across Europe (21) and the world (22), which has been intensely actualized in the context of the covid-19 pandemic [e.g., (23)]. Vaccine hesitancy has been tied to contemporary contestation of expert knowledge (22, 24) or to a current “war on science” (25) as well as to the internet today being an important source of health-related information. The latter has generated concern about negative views of vaccines spreading on Facebook and other social media (26–29). Such communication of vaccine hesitant views is often understood as being a matter of misinformation (30, 31), misunderstanding of science (32) or a public knowledge deficit (25, 33) in what has been called a post-truth era (34). Some scholars point out, however, that vaccine hesitancy, the history of which is not isolated to recent decades (35, 36), is a complex phenomenon comprising sociocultural, political and psychological factors (25, 36, 37).

In a systematic review of qualitative studies on parents' views of HPV vaccination, Marshall et al. (10) note that degrees of vaccine hesitancy were evident in all the studied populations. In a Swedish survey study conducted in 2007, i.e., before the initiation of the free-of-charge, school-based HPV vaccination program (38), 24% of the responding parents were unwilling (3%) or unsure (20%) about whether to let their children be vaccinated. While low income, low education and migration background have been associated with lower levels of vaccination uptake (17–19), vaccine hesitancy was here associated with higher education and with being in employment (38). In a smaller Danish survey from 2016 (11), 34% of parents were hesitant toward HPV vaccination. Meanwhile, studies of HPV vaccine attitudes and decision-making processes among parents and young people in Sweden (39–42) and elsewhere (10, 21, 43–47) point to complex processes leading to the choice to vaccinate, or to not (10, 42, 45), on the basis of the perceived benefits and risks of the vaccine (42, 48).

A factor often found to influence decision-making is concerns for vaccine safety or side effects (10, 21, 38, 39, 42, 43, 46, 47, 49), which include references to the relative newness of the HPV vaccine (10, 46). While scientific studies support the safety of the HPV vaccine (50–53), perceived side effects noted in international qualitative studies include infertility, autoimmune complications, cancer and death (21). In studies from Sweden, concerns related to the side effects of the H1N1 (or swine flu) vaccine Pandemrix, which was administered in 2009–2010 and later linked to over 200 cases of the neurological disease narcolepsy among children and young adults (54, 55), were raised both by parents who had decided to vaccinate their child/ren (39) and by parents who had not (42). Studies have also pointed to participants being unsure about the efficacy of the HPV vaccine (38, 46).

Vaccine hesitancy has been linked to a lack of trust not only the vaccines themselves but also in the institutions that produce and administer them (25, 56–58). General levels of trust in vaccinations, healthcare providers and governments, alongside worries about commercial interests associated with the pharmaceutical industry, have thus been found to reportedly affect HPV vaccine decisions in Sweden (39, 42) as in other countries (10, 21, 44, 45, 56–58). A more general distrust in official information and governmental recommendations observed in a study of parents who had declined HPV vaccination (42) was noted by the authors to represent a relatively new phenomenon in the Swedish context.

The perceived presence or absence of sufficient knowledge or information about HPV and the vaccine has furthermore been noted to affect decision-making (10, 21, 39, 43), and was pointed out as a key reason for declining HPV vaccination in a study from Sweden (42). Fear of, or personal experience with, HPV related illness has been noted to affect decision-making, as has the perceived risk of HPV related disease (10, 42, 43, 46). Communication with healthcare providers (10, 43, 46) and the practices and attitudes of school nurses (39, 59) have also been noted to play a part. Other factors noted to influence decision-making include thinking the recipients were too young (42, 43, 60), concern that the vaccination may be conducive to an early sexual debut, to having many sexual partners, or to being less attentive to contraceptives and pap smears due to a false sense of security (10, 21, 42).

The noted studies, some of which were conducted in Sweden (38, 39, 42), have increased the understanding of attitudes toward and decision-making processes surrounding HPV vaccination. There is, however, a paucity of recent research on attitudes toward the HPV vaccine in a larger sample of the population in Sweden (38), although updated knowledge is highly relevant due to the mentioned trends in information-seeking, to the current actualization of the issue of vaccine hesitancy, and to changes in the HPV vaccination program which now also includes boys. The aim of this study is therefore to assess attitudes toward HPV-vaccination in the Swedish population, including any correlations between vaccine hesitancy and sociodemographic characteristics, trust in healthcare and other societal institutions, and evaluation of the reliability of different sources of information. Because mothers often make or strongly influence decisions regarding HPV vaccination (41, 43, 45), since a large share of persons sharing vaccine-negative views in social media are women (29, 61), and since this study is part of a larger project concerning women's health issues, the survey was targeted to women in Sweden.

## Materials and Methods

This nation-wide survey study targeted adult women (18–55 years, *n* = 2,000) in Sweden, and was conducted in collaboration with the online market research company Kantar Sifo (www.kantarsifo.se). Respondents were randomly selected from Kantar Sifo's web panel, which consists of around 100,000 active panelists recruited through nationally representative random selection (62).

The questionnaire was developed by the researchers, in collaboration with Kantar Sifo, and included some previously used survey items (63) and some items specifically developed for the current study. Questions were posed about the likelihood that the respondents would let their daughter or son receive the HPV vaccination, and, inspired by the Health Belief Model (64), measured the respondents' assessment of the benefits and, if any, risks of HPV vaccination. The respondents were also asked to evaluate the credibility of different sources of information concerning HPV vaccination, and to rate their general trust in a range of societal institutions. Sociodemographic information, beyond that already assembled by Kantar Sifo, was also gathered. Most questions were closed, enabling quantitative analysis, while one open question enabled the respondents to express themselves more freely. Apart from the questions regarding attitudes toward the HPV-vaccine, addressed in this article, the questionnaire also inquired about the respondents' attitudes toward the contraceptive copper intrauterine device (IUD). The survey, excluding the questions concerning the copper IUD, is found in the Appendix.

The questionnaire was validated through review by three healthcare professionals with research experience and through a pilot launch in the Kantar Sifo web panel (*n* = 100) including open questions about the clarity and relevance of the survey questions. The data gathering was conducted from Dec 17 2020 to Jan 8 2021. The survey was distributed *via* email, and completed *via* computer, tablet or mobile phone. Information about the study was included in the email, and consent was given through completion of the survey. Participants who had not yet responded to the survey after a number of days received 1–2 reminders.

The number of respondents was set at 2000, to allow for some sub-group analysis. The response rate was 37%, which approximates the average response rates of web panels conducted by Kantar Sifo. In order to improve representativity, the results were weighted with regard to age and region, according to national weights provided by Statistics Sweden. The quality control performed by Kantar Sifo, including response time and straight-liner analyses (65), deemed the data to be of good quality. The raw data delivered to the research group by Kantar Sifo was completely anonymized.

The project was approved by the Swedish Ethical Review Authority (Dnr: 2019-03017).

### Data Analysis

Apart from simple descriptive statistics, multiple logistic regressions were used to assess any correlations between vaccine hesitancy and sociodemographic and other variables. Associations were expressed as odds ratios (ORs) with 95% confidence intervals (95% CIs). The statistical analyses were made using IBM SPSS Version 27.0 (SPSS Inc., Chicago, IL, USA). The open survey responses were analyzed thematically (66), i.e., divided into themes and categories, with the aid of the software program NVivo.

### Assessment of Variables

In the regression analyses, two variables (VH1; VH2) were used as indicators of HPV vaccine hesitancy. These were based on the survey questions: “If you have, or were to have, a daughter/son [respectively] who was offered HPV vaccination, how likely do you perceive it to be that you would let the child receive the vaccine?,” offering the response options *very likely*; *rather likely*; *rather unlikely*; *very unlikely*; and *unsure*. Having responded something other than *very likely* to either of these two questions was used as an indicator of some degree of HPV vaccine hesitancy (VH1). Having responded that HPV vaccination receipt was *rather unlikely*; *very unlikely*; or *unsure* with regard to a daughter was used as an indicator of more pronounced HPV vaccine hesitancy (VH2). This was done to distinguish a more pronounced vaccine hesitancy from a less clear one, and to exclude hesitation perhaps directly related to the quite recent targeting of boys.

Due to the large number of variables and analyses, the remaining variables will be described in association with the results of the respective analyses.

## Results

Characteristics of the study population can be seen in Table 1.

**Table 1:** Study population characteristics and (unweighted) distribution of vaccine hesitancy between sociodemographic groups.

	VH1^*^		VH2^**^	
	**Hesitancy**	**No hesitancy**	**Hesitancy**	**No hesitancy**
	***n* (%)**	***n* (%)**	***n* (%)**	***n* (%)**
Women	671 (33.6)	1,324 (66.4)	163 (8.2)	1,835 (91.8)
Other gender identity (with womb)	0	2 (100)	0	2 (100)
18–29 years	165 (39.1)	257 (60.9)	18 (4.3)	404 (95.7)
30–49 years	346 (32.3)	726 (67.7)	100 (9.3)	974 (90.7)
50–64 years	160 (31.8)	343 (68.2)	45 (8.9)	459 (91.1)
Tertiary education	431 (29.4)	1,033 (70.6)	87 (5.9)	1,379 (94.1)
Less than tertiary education	237 (44.8)	292 (55.2)	75 (14.2)	455 (85.8)
High income	83 (23.1)	277 (76.9)	17 (4.7)	344 (95.3)
Medium income	322 (32.3)	676 (67.7)	70 (7.0)	929 (93.0)
Low income	207 (41.3)	294 (58.7)	60 (12.0)	442 (88.0)
Born in Sweden	645 (33.6)	1,275 (66.4)	157 (8.2)	1,766 (91.8)
Born elsewhere	26 (34.7)	49 (65.3)	6 (8.0)	69 (92.0)
Children	376 (30.9)	840 (69.1)	106 (8.7)	1,112 (91.3)
No children	295 (37.8)	486 (62.2)	57 (7.3)	725 (92.7)

* *VH1 distinguishes between those reporting it to be very likely (no hesitancy) that they would let their daughter or son receive the HPV vaccine from those reporting it to be rather likely, rather unlikely, very unlikely, or that they did not know (hesitancy). This indicates some HPV vaccine hesitancy*.

** *VH2 distinguishes between those reporting it to be very or rather likely (no hesitancy) that they would let their daughter receive the HPV vaccine from those reporting it to be rather or very unlikely, or that they did not know (hesitancy). This indicates more pronounced HPV vaccine hesitancy*.

### Attitudes Toward Vaccination

To the question about the likelihood of letting a daughter or son receive the HPV vaccine, a majority reported this to be very likely: 79.1% in the case of a daughter and 66.4% in the case of a son (Table 2). With reference to daughters, 7.6% (*n* = 153) responded that consenting to HPV vaccination was rather or very unlikely, or that they did not know. These respondents were grouped in the pronounced HPV vaccination hesitancy variable (VH2). The corresponding figure regarding boys was 18.9%. The share who responded something other than very likely on either question, thus indicating some degree of HPV vaccine hesitancy (VH1) was 33.8% (*n* = 675).

**Table 2:** Attitudes toward the HPV vaccine.

**If you have, or were to have, a girl/boy child who was offered HPV vaccination, how likely do you perceive it to be that you would let the child receive the vaccine?**	**Girl *n* (%)**	**Boy *n* (%)**
Very likely	1,582 (79.1)	1,329 (66.4)
Rather likely	265 (13.2)^*^	292 (14.6)^*^
Rather unlikely	40 (2.0)^**^	65 (3.3)^*^
Very unlikely	47 (2.4)^**^	75 (3.8)^*^
Unsure or unwilling to respond	66 (3.3)^**^	236 (11.8)^*^
Vaccine hesitancy 1 (VH1)^*^	675 (33.8)	
Vaccine hesitancy 2 (VH2)^**^	153 (7.6)	

* *VH1 distinguishes between those reporting it to be very likely (no hesitancy) that they would let their daughter or son receive the HPV vaccine from those reporting it to be rather likely, rather unlikely, very unlikely, or that they did not know (hesitancy). This indicates some HPV vaccine hesitancy*.

** *VH2 distinguishes between those reporting it to be very or rather likely (no hesitancy) that they would let their daughter receive the HPV vaccine from those reporting it to be rather or very unlikely, or that they did not know (hesitancy). This indicates more pronounced HPV vaccine hesitancy*.

In response to questions about the perceived benefits and risks of HPV vaccination (Table 3), between half and two-thirds of the respondents reported being in strong agreement that HPV vaccination fills an important function in preventing cervical cancer (65.8%), that it provides effective protection against cervical cancer (50.7%) and that the benefits of HPV vaccination outweigh its potential risks (52.9%). Around half reported agreeing strongly or fairly strongly that any risks or side effects of the HPV vaccine are uncommon (56.8%) and mild (49.4%). While a small share responded that they disagreed or agreed to a fairly low degree to these latter two questions (4.4 and 4.7%), a larger number reported being unsure (38.4 and 45.4%). 15.4% responded being unsure whether the benefits of HPV vaccination outweigh its potential risks.

**Table 3:** Perceived risks and benefits of the HPV vaccine, and associations between HPV vaccine hesitancy (VH1; VH2) and less or no agreement with the respective statements, measured through logistic regressions and expressed as odds ratios (ORs) with 95% confidence intervals (CIs).

	**Agree to high degree *n* (%)**	**Agree to fairly high degree *n* (%)**	**Agree to fairly low degree *n* (%)**	**Disagree *n* (%)**	**Unsure *n* (%)**	VH1^*^OR (95% CI)	VH2^**^OR (95% CI)
The HPV vaccine fills an important function in preventing cervical cancer	1,315 (65.8)	485 (24.3)	35 (1.8)	23 (1.2)	135 (6.8)	9.41 (7.60–11.65)	52.91 (24.23–115.53)
The HPV vaccine provides effective protection against cervical cancer	1,013 (50.7)	642 (32.1)	58 (2.9)	17 (0.9)	263 (13.1)	6.27 (5.07–7.76)	40.40 (15.61–104.61)
The risks and side effects of the HPV vaccine are uncommon	611 (30.5)	525 (26.3)	56 (2.8)	31 (1.6)	767 (38.4)	5.57 (4.28–7.27)	13.35 (5.62–31.74)
The risks and side effects of the HPV vaccine are mild	509 (25.5)	480 (24.0)	61 (3.1)	33 (1.6)	908 (45.4)	5.68 (4.23–7.63)	24.27 (6.65–88.57)
The benefits of the HPV vaccine outweigh its potential risks	1,057 (52.9)	540 (27.0)	50 (2.5)	40 (2.0)	307 (15.4)	7.32 (5.91–9.07)	64.56 (20.81–200.35)

* *VH1 distinguishes between those reporting it to be very likely (no hesitancy) that they would let their daughter or son receive the HPV vaccine from those reporting it to be rather likely, rather unlikely, very unlikely, or that they did not know (hesitancy). This indicates some HPV vaccine hesitancy*.

** *VH2 distinguishes between those reporting it to be very or rather likely (no hesitancy) that they would let their daughter receive the HPV vaccine from those reporting it to be rather or very unlikely, or that they did not know (hesitancy). This indicates more pronounced HPV vaccine hesitancy*.

The questions about the perceived benefits and risks of the HPV vaccine were dichotomized, for the purpose of the logistic regression analyses, by dividing those reporting being in strong agreement from those responding something else (*strong agreement* vs. *agreeing less; disagreeing; unsure*). Not being in strong agreement was strongly associated with some degree of vaccine hesitancy, and more so with pronounced vaccine hesitancy. For example, not being in strong agreement that the HPV vaccine provides effective protection of cervical cancer was highly associated with vaccine hesitancy [VH1: OR 6.27 (95% CI 5.07–7.76); VH2: OR 40.40 (95% CI 15.61–104.61)], as was not being in strong agreement that the risks and side effects of the HPV vaccine are uncommon [VH1: OR 5.57 (95% CI 4.28–7.27); VH2: OR 13.35 (95% CI 5.62–31.74)] and mild [VH1: OR 5.68 (95% CI 4.23–7.63); VH2: OR 24.27 (95% CI 6.65–88.57)].

An additional question inquired about the respondents' attitude toward vaccination in general. Just over half, 55.8%, indicated a very positive attitude, and 36.9% a fairly positive attitude. The remaining 7.4% indicated that they were fairly or very negative, or unsure. Regression analysis using the dichotomized variable (*very positive* vs. *less positive, negative or unsure*) showed that being less than very positive about vaccination in general was strongly associated with HPV vaccine hesitancy [VH1: OR 4.38 (95% CI 3.59–5.34); VH2: OR 11.54 (95% CI 6.93–19.21)] (**Table 5**).

### Sociodemographic Factors

As seen in Table 1, which presents unweighted data on HPV vaccine hesitancy in the study population, HPV vaccine hesitancy was more common among those with lower education and lower income. While some degree of HPV vaccine hesitance (VH1) was most prevalent in the youngest age group, more pronounced hesitancy (VH2) was more common in older age groups. Vaccine hesitancy (VH1) was more common among those without children, while more pronounced hesitancy (VH2) was slightly more prevalent among those with children.

With regard to the sociodemographic variables, education was categorized as having a tertiary education or not (*high* vs. *low* education). The personal income variable used and conflated categories previously used by Kantar Sifo, and distinguished between those with a monthly income of SEK < 24,999 (*low* income), SEK 25,000–41,999 (*medium* income) and SEK 41,999 < (*high* income) (SEK 10,000 = USD ~1,000). The age variable divided the respondents into three age groups (*18*–*29*; *30*–*49*; *50*–*64* years). The country of birth variable distinguished between those born in Sweden or in another country (*Sweden* vs. *elsewhere*). The parenthood variable distinguished between having (co-habiting or non-cohabiting) children or not (*children* vs. *no children*).

Any correlations between vaccine hesitancy and each of these variables were measured individually, and those variables showing conclusive associations were entered jointly in a new regression model. The associations which remained conclusive are shown in Table 4.

**Table 4:** Associations between sociodemographic characteristics and HPV vaccine hesitancy (VH1; VH2), expressed as odds ratios (ORs) with 95% confidence intervals (CIs).

	VH1^*^OR (95% CI)	VH2^**^OR (95% CI)	Vaccines generally^***^OR (95% CI)
**Education**			
High	Reference	Reference	Reference
Low	1.51 (1.20–1.90)	2.10 (1.41–3.13)	1.28 (1.02–1.61)
**Income**			
High	Reference	Reference	Reference
Medium	1.48 (1.10–2.00)	Inconclusive	1.76 (1.34–2.32)
Low	1.74 (1.26–2.42)	2.94 (1.54–5.63)	2.04 (1.47–2.84)
**Age**			
18–29	Reference	Reference	Reference
30–49	Inconclusive	3.95 (2.36–6.59)	1.37 (1.08–1.73)
50–64	Inconclusive	3.71 (2.05–6.72)	1.89 (1.41–2.54)

* *VH1 distinguishes between those reporting it to be very likely (no hesitancy) that they would let their daughter or son receive the HPV vaccine from those reporting it to be rather likely, rather unlikely, very unlikely, or that they did not know (hesitancy). This indicates some HPV vaccine hesitancy*.

** *VH2 distinguishes between those reporting it to be very or rather likely (no hesitancy) that they would let their daughter receive the HPV vaccine from those reporting it to be rather or very unlikely, or that they did not know (hesitancy). This indicates more pronounced HPV vaccine hesitancy*.

*** *This variable distinguishes between those reporting a very positive attitude (no hesitancy) toward vaccination in general from those reporting a fairly or less positive or negative attitude, or being unsure (hesitancy)*.

The correlation with lower education was stronger for VH2 [OR 2.10 (95% CI 1.41–3.13)] than for VH1 [OR 1.51 (95% CI 1.20–1.90)]. Similarly, the association with low income was stronger for VH2 [OR 2.94 (95% CI 1.54–5.63)] than for VH1 [OR 1.74 (95% CI 1.26–2.42) for VH1]. Regarding VH1, an income gradient was suggested. Pronounced vaccine hesitancy (VH2) was more common among those aged 30–49 years [OR 3.95 (95% CI 2.36–6.59)] or 50–64 years [OR 3.71 (95% CI 2.05–6.72)], compared to those aged 18–29 years. No conclusive associations were identified based on parenthood or country of birth.

Concerning attitudes toward vaccines overall, a less positive attitude was associated with lower education [OR 1.28 (95% CI 1.02–1.61)], lower income [OR 1.76 (95% CI 1.34–2.32)]; [OR 2.04 (95% CI 1.47–2.84)] and older age [OR 1.37 (95% CI 1.08–1.73); OR 1.89 (95% CI 1.41–2.54)].

### Evaluation of Information and Healthcare

The respondents were asked to rate their general confidence in a number of societal institutions, and in sources of information relating specifically to the HPV vaccine. Additional questions inquired about the evaluation of information about, and assessments of healthcare in relation to, the HPV vaccine (Table 5).

**Table 5:** Associations between attitudes regarding information and healthcare and HPV vaccine hesitancy (VH1; VH2), expressed as odds ratios (ORs) with 95% confidence intervals (CIs).

	VH1^*^OR (95% CI)	VH2^**^OR (95% CI)
**General**		
Healthcare	2.05 (1.55–2.70)	4.53 (3.12–6.56)
Researchers	3.26 (2.39–4.45)	8.93 (6.15–12.97)
Politicians	1.62 (1.33–1.98)	2.35 (1.57–3.53)
School	1.51 (1.23–1.85)	2.46 (1.76–3.45)
Social insurance agency	1.34 (1.10–1.63)	1.71 (1.18–2.46)
Police	1.44 (1.13–1.84)	2.28 (1.57–3.31)
**Regarding HPV–vaccine**		
Medical professionals	6.16 (4.28–8.88)	16.94 (11.53–24.88)
Regulatory institutions (LMV, SOS)	4.56 (3.34–6.21)	14.69 (10.18–21.19)
CAM	0.67 (0.53–0.87)	0.51 (0.35–0.76)
Social media	Inconclusive	Inconclusive
Other people	Inconclusive	Inconclusive
Own experiences	1.39 (1.14–1.70)	2.18 (1.55–3.06)
**Evaluation of information**		
Source criticism	1.57 (1.31–1.90)	inconclusive
Access to information	3.72 (2.82–4.91)	3.52 (1.91–6.49)
Easy to discern origin of information	2.94 (2.17–3.98)	3.85 (1.88–7.90)
Ability to assess quality and reliability of information	3.05 (2.28–4.06)	2.78 (1.53–5.07)
**Having taken part in info/stories on risks associated with HPV-vaccine**		
From healthcare	Inconclusive	Inconclusive
From CAM	1.83 (1.17–2.86)	4.67 (2.75–7.92)
From other individuals	1.39 (1.05–1.85)	3.06 (2.06–4.53)
Never	0.76 (0.62–0.93)	0.43 (0.31–0.60)
**Healthcare**		
Satisfied with healthcare	1.67 (1.05–2.66)	7.30 (3.22–16.56)
**Vaccination**		
General vaccine hesitancy	4.38 (3.59–5.34)	11.54 (6.93–19.21)

* *VH1 distinguishes between those reporting it to be very likely (no hesitancy) that they would let their daughter or son receive the HPV vaccine from those reporting it to be rather likely, rather unlikely, very unlikely, or that they did not know (hesitancy). This indicates some HPV vaccine hesitancy*.

** *VH2 distinguishes between those reporting it to be very or rather likely (no hesitancy) that they would let their daughter receive the HPV vaccine from those reporting it to be rather or very unlikely, or that they did not know (hesitancy). This indicates more pronounced HPV vaccine hesitancy*.

Regarding societal institutions, the respondents were asked to rate their general confidence in healthcare, researchers, politicians, schools, the social insurance agency and the police, on a four-point scale (*very strong*; *rather strong*; *rather weak*; *very weak*). Overall, very or rather strong confidence was reported by 88.4% in relation to healthcare, 90.6% in the case of researchers, 36.2% for politicians, 74.0% for schools, 38.8% for the social insurance agency, and 83.9% for the police.

For the logistic regression analyses, the variables were dichotomized (*very strong; rather strong* vs. *rather weak; very weak*). Vaccine hesitancy was associated with a weaker level of confidence in all these institutions, and the association was consistently stronger for the VH2 variable than for VH1 (Table 5). Notably, confidence was particularly low among vaccination hesitant respondents for healthcare [VH1: OR 2.05 (95% CI 1.55–2.70); VH2: OR 4.53 (95% CI 3.12–6.56)] and researchers [VH1: OR 3.26 (95% CI 2.39–4.45); VH2: OR 8.93 (95% CI 6.15–12.97)].

Furthermore, the respondents were asked to assess the trustworthiness of different sources of information relating specifically to the HPV vaccine, on a four-point scale (*very reliable; rather reliable; rather unreliable; very unreliable*). The sources were medical professionals including doctors and nurses; institutions regulating medical practice, such as the Medical Products Agency (Läkemedelsverket, LMV) and the National Board of Health and Welfare (Socialstyrelsen, SOS); complementary and alternative medicine (CAM) sources; social media; the stories of other individuals who the respondent had met; and one's own experiences. Overall, medical professionals were regarded as very or rather reliable by 91.8%, regulatory institutions by 89.5%, CAM sources by 15.3%, social media by 4.9%, the stories of other individuals by 50.4% and one's own experiences by 68.0%.

For the regression analyses, the variables were dichotomized (*very reliable; rather reliable* vs. *rather unreliable; very unreliable*). Vaccine hesitancy was strongly associated with assessments of medical professionals [VH1: OR 6.16 (95% CI 4.28–8.88); VH2: OR 16.94 (95% CI 11.53–24.88)] and regulatory institutions [VH1: OR 4.56 (95% CI 3.34–6.21); VH2: OR 14.69 (95% CI 10.18–21.19)] as being less reliable, compared to those reporting no hesitancy. Moreover, vaccine hesitancy was associated with regarding CAM sources as more reliable [VH1: OR 0.67 (95% CI 0.53–0.87); VH2: OR 0.51 (95% CI 0.35–0.76)], although the correlation was weaker than the prior ones [for the purpose of comparability, if the association was measured in reverse to yield a positive correlation, VH1: OR 1.48 (95% CI 1.16–1.90); VH2: OR 1.95 (95% CI 1.32–2.89)]. With regard to social media, or to the stories of individuals that respondent had met, no conclusive associations were identified. Own experiences were regarded as less reliable by those reporting vaccine hesitancy.

Additional questions inquired about whether the respondents had taken part of information or stories about any risks pertaining to the HPV vaccine from healthcare professionals, from CAM sources, or from other individuals. Regarding information from healthcare, no conclusive difference was identified between those expressing vaccine hesitancy or not. Vaccine hesitancy was, meanwhile, associated with having taken part of information or stories from CAM sources [VH1: OR 1.83 (95% CI 1.17–2.86); VH2: OR 4.67 (95% CI 2.75–7.92)] or from other individuals [VH1: OR 1.39 (95% CI 1.05–1.85); VH2: OR 3.06 (95% CI 2.06–4.53)]. Not having taken part in any information or stories at all about risks related to the vaccine was more common among those not reporting vaccine hesitancy [VH1: OR 0.76 (95% CI 0.62–0.93); VH2: OR 0.43 (95% CI 0.31–0.60)].

Respondents were also asked about the evaluation of information. One question inquired about general self-assessed source criticism, by asking about the degree to which the respondent agreed with the statement “It is generally important for me to be source critical and find out where health-related information comes from.” With specific reference to the HPV vaccine, respondents were asked about the degree to which they agreed that they had access to the information they needed about the vaccine, that they found it easy to discern where information about the HPV vaccine comes from, and that they had the ability to assess the quality and reliability of different kinds of information about the vaccine. The response options consisted of a five-point scale (*agree to a high degree; agree to a fairly high degree; agree to a fairly low degree; agree to a low degree or not at all; unsure*), with the additional option in the latter three questions of responding that information about the HPV vaccine had not been sought. The approximate one-third of the respondents who chose the latter option were not included in the logistic regression analyses. Overall, strong agreement about the general importance of source criticism regarding health-related information was reported by 57.2%, while 38.7% reported being in fairly strong agreement. When those who reported not having sought information about the HPV vaccine were excluded, 40.2% were in strong agreement that they had access to sufficient information about the HPV vaccine, 29.5% that they found it easy to discern where information about the HPV vaccine came from and 32.0% that they had the ability to assess the quality and reliability of information about the vaccine.

The variables were dichotomized for the logistic regressions (*agree strongly;* vs. *agree somewhat; disagree; unsure*). Agreeing less or disagreeing about the importance of source criticism was more common among those reporting some degree of HPV vaccine hesitance [VH1: OR 1.57 (95% CI 1.31–1.90)]. No conclusive association was identified with more pronounced HPV vaccine hesitancy (VH2). Regarding information about the HPV vaccine, agreeing less, disagreeing or being unsure about the latter three statements was more common among those reporting vaccine hesitancy. For example, less agreement with having access to sufficient information was strongly associated with HPV vaccine hesitancy [VH1: OR 3.72 (95% CI 2.82–4.91); VH2: OR 3.52 (95% CI 1.91–6.49)].

Finally, 28.2% (*n* = 563) of the respondents reported having been in contact with healthcare personnel concerning HPV vaccination. Of these, 85% (*n* = 536) reported being very (53%) or rather (32.1%) satisfied with those health care encounters. Being dissatisfied or unsure (*very satisfied; rather satisfied* vs. *rather dissatisfied; very dissatisfied; unsure*) was associated with HPV vaccine hesitancy [VH1: OR 1.67 (95% CI 1.05–2.66); VH2: OR 7.30 (95% CI 3.22–16.56)].

### Open Text Responses

The survey included an open question asking if the respondent wanted to add any comment regarding HPV vaccination. After the exclusion of non-indicative responses such as “no” and “no comment,” this question yielded 320 responses. These were divided, based on their content, into three themes: *attitudes toward HPV vaccination, information about the vaccination*, and *offering the HPV vaccination to boys* (Table 6).

**Table 6:** Open survey responses: themes and categories.

**Themes**	**Categories**	**Sub-categories**
Attitudes toward HPV vaccination (*n* = 320)	Positive	
	Value-neutral	
	Ambivalent or negative	Negative
		Processes of learning/negotiation
Information about the vaccination (*n* = 91)	Active process	
	Need for information	
Offering HPV vaccination to boys (*n* = 35)		

#### Attitudes Toward HPV Vaccination

With regard to attitudes toward HPV vaccination, all responses (*n* = 320) were divided into positive, value-neutral and ambivalent or negative comments. Most (*n* = 192) were included in the *positive* category, which contained comments about having received the vaccine, for oneself or with reference to a child, and positive statements about the vaccine. Some respondents (*n* = 11) used the words “self-evident,” “natural,” or “definitely” with reference to the decision to receive the HPV vaccination. Several referred to not being vaccinated, most commonly due to being too old, but wishing that they had been, with some indicating experiences of HPV related disease. A couple of respondents pointed to a decrease in cervical cancer due to HPV vaccination, and one commented that the public attitude toward the HPV vaccine is generally positive in Sweden:

Feels very socially accepted (R87).

The *value-neutral* responses (*n* = 84) included comments such as being too old and thus ineligible for the vaccination or not knowing very much about it.

Of the *ambivalent or negative* (*n* = 75) responses, a smaller share (*n* = 13) were categorized as *negative*. These contained statements about not having let children receive the vaccination, alongside references to claimed side effects and insufficient research on the vaccine's long-term effects. Many of the remaining responses comprised a sub-category pointing to *processes of learning or negotiating*, as they referred to changing or ambivalent view-points on the vaccine and its risks and benefits. Some wrote about not having received the vaccine, due to their own or their parents' skepticism, but now wishing they had. Others commented on having let their children receive the vaccine but now regretting it or being unsure if it was the right decision.

I didn't let my daughter take the vaccine when it was offered at school because I didn't have enough information and I didn't want to risk anything for my daughter. Now 5 years later I have read studies around the vaccine and feel safe about her taking it, when this [COVID-19] pandemic is over (R276).

Our daughter took the vaccine before “everyone” got it and we paid for it ourselves. I realize afterwards that I should have read more about it—even if we paid ourselves we didn't go down deep to really check that it was an OK vaccine. Today I think that was quite naïve (R152).

Several expressed a concern for potential side effects.

We vaccinated our daughter, just before it became obligatory. Are afraid that her chronic headache came through Gardasil. Have no evidence other than the point in time and the result (R91).

A few referred to the side effects of the H1N1 flu vaccine as having had some form of impact on their opinions.

Vaccines were generally “OK” until the swine flu—then one became more skeptical (R44).

Some commented on pap smears and contraceptives (condoms) being other or more important forms of cancer prevention, and on the HPV vaccine as potentially creating a false sense of security. One respondent referred to the importance of other means of preventing disease, such as maintaining a clean and sustainable environment. Differences between views on or debates about side effects in different countries were referred to. Fear of needles was mentioned as an additional reason for not wanting to receive the vaccine.

#### Information About the Vaccination

Almost one-third of the responses (*n* = 91) included references to information or knowledge about the HPV vaccine. Some spoke about being satisfied with existing information, or not having taken part of information e.g., due to not being, or not having a child who was, eligible for HPV vaccination.

In accordance with the sub-category named “processes of learning or negotiating,” above, a large share of the comments here referred to information seeking and evaluation as an *active process*. Respondents commented on having received or sought information from research studies, healthcare personnel, other individuals and social media, alongside personal experiences.

My daughter is vaccinated but because I was skeptical, due to side effects, I did some research beforehand (R290).

I've been more negative before but after the most recent follow-up was published, I am more positive (R82).

Correspondingly, many comments expressed a *need of more information*. Some referred to a lack of information having contributed to vaccination non-receipt.

I wish I had taken it before I had sex. Insufficient information was given to those who were skeptical (i.e., me) explaining how the vaccine worked (R90).

As I remember, we hardly received any information at all at school and I thought all vaccines had a risk for serious side effects since the vaccine for swine flu did (R10).

Others commented on wanting to see more research on the subject, including on the long-term effects of the vaccine, and clearer information about the risk of cervical cancer vs. risks of vaccine side effects. A need to improve information from and communication by healthcare professionals was also expressed.

I think the information sent out by the school in connection to my daughter's vaccination was very unpleasant. I wanted to vaccinate my child but don't like that they have the tone “if you don't vaccinate the child it will die in cervical cancer.” That's not how we make people choose to vaccinate. Concrete data. Descriptions. Explanations. Risk percentages, for and against! That's what it should be about (R130).

One comment referred to a lack of information from healthcare institutions being conducive to people turning to alternative sources and to vaccine hesitancy.

When it comes to all kinds of vaccines, healthcare must become better at communicating the meaning of vaccination. Otherwise, people will turn to alternative sources, which may cause increased vaccine skepticism (R288).

#### Offering the HPV Vaccination to Boys

Several comments (*n* = 35) referred to the HPV vaccination being offered to boys. A minority expressed doubt about the benefit for boys, while most were positive about the inclusion of males in the vaccination program due to contagion and the risk of HPV related cancers also afflicting men.

## Discussion

The results of this survey study point, on the one hand and as expected, to a positive attitude toward HPV vaccination in Sweden. The vast majority, 92.4% of the respondents, reported it to be very or rather likely that they would let their daughter receive the HPV vaccination, and 81.0% in the case of a son. Most open text responses expressed a positive attitude, some using words such as “self-evident” or “definitely” when referring to HPV vaccination. Similarly, 93% reported a positive attitude toward vaccinations in general.

On the other hand, the results point to attitudes not being entirely positive. The share of respondents reporting something other than it being very likely that they would let their child receive the HPV vaccination, and who were thus included in the group indicating some degree of HPV vaccine hesitancy (VH1), was 33.8%. Furthermore, when asked about the perceived benefits and risks of the HPV vaccine, only around half of the respondents (52.9%) reported being in strong agreement that the benefits of the HPV outweigh its potential risks, and being in strong or fairly strong agreement that its side effects are uncommon (56.8%) and mild (49.5%). A large share of the remaining respondents reported being unsure. Moreover, only just over half (55.8%) indicated a very positive attitude toward vaccination in general. In addition, as seen in the open text responses, rather than simply accepting the HPV vaccination as recommended through the national vaccination program, many respondents described an active process of seeking, receiving and evaluating information about the vaccine, and to sometimes changing their minds over time. In this sense, our results are in line with Larson's [(67), p. 1,207] understanding of the concept of vaccine hesitancy as “depolarizing the earlier characterization of individuals or groups as being outright pro- or antivaccine, and instead recognizing the liminal state between becoming aware of, and deciding whether or not to accept, vaccination,” as well as with Hausman's (36) argument that in some contrast to common and polarizing representations of vaccine hesitancy, such hesitancy can be seen as expressions of concerns that are not very uncommon in the population. This result can also be related to research indicating higher levels of vaccine hesitant *attitudes* than of non-vaccination *behaviors* in European and North American countries (25).

### Socioeconomic Factors

Vaccine hesitancy was found to be associated with sociodemographic factors. Some degree of HPV vaccine hesitancy (VH1), and particularly pronounced hesitancy (VH2), were associated with lower education. Vaccine hesitancy was also more common among those with low and, in the case of some degree of HPV vaccine hesitancy (VH1), medium income. These results differ from a previous survey study of attitudes toward HPV vaccination in Sweden (38), which found vaccine hesitancy to be associated to higher education and being in paid employment (45, 68). Other studies have also observed a stronger presence of vaccine hesitancy in more privileged groups and countries (25, 69). The noted study on HPV vaccination in Sweden (38) was conducted in 2007, before the introduction of the school-based, free-of-charge HPV vaccination program, and the association has been interpreted (18) as likely being due to highly educated parents being more cautious regarding a recently introduced vaccine. While this may be so, today vaccine hesitancy is often associated with populist movements (70) positioning themselves against a perceived elite and therefore possibly speaking largely to groups with lower socioeconomic status (71). Meanwhile, the understanding of vaccine hesitancy as being more prevalent in more affluent groups has been posited as resulting from limited research (25). In line with our results, and with other studies showing lower vaccination rates (14, 16, 72) and stronger vaccine hesitancy (73, 74) in less privileged groups, HPV vaccination uptake in Sweden has been lower among girls whose parents have a lower education or income (17–19). In the current study, pronounced vaccine hesitancy (VH2) was furthermore more common in older age groups, while no conclusive difference between age groups was identified with regard to some degree of hesitancy (VH1). No conclusive difference was found between those with or without children, or between those born in Sweden or elsewhere. That said, it should be noted that the participants reporting being born in another country were very few in number (*n* = 75).

### Information, Decision-Making, and Trust

Vaccine hesitancy was associated with agreeing less or not at all about having access to sufficient information about HPV vaccination, about finding it easy to discern where information about the HPV vaccines come from, and about having the ability to assess the quality and reliability of information about the vaccine. Meanwhile, many of the open text responses pointed to a need for more and clearer information about HPV vaccination, communicated by healthcare professionals and institutions, and to respondents actively seeking and evaluating information from different sources.

This points to the importance of providing clear and nuanced information about the HPV vaccination to prospective recipients and their caregivers. That is in accordance with research noting caregivers' active information seeking and self-assessed lack of sufficient information about the HPV vaccine (42), requests for more information about the HPV vaccine (28), and limited knowledge or information being an important contributing factor to HPV non-vaccination (10, 21, 39, 42). These results are also in line with research indicating that decision-making regarding HPV vaccination (39, 42), and other vaccinations (26), can entail complex processes during which various sources of information are considered.

At the same time, however, vaccine hesitancy was associated, sometimes very strongly so, with a lower level of general confidence in societal institutions, particularly in healthcare and in researchers, and in the reliability of medical professionals and regulatory institutions specifically regarding the HPV vaccine. Some degree of vaccine hesitancy, although not its more pronounced variety (VH2), was, meanwhile, associated with a lower degree of self-assessed source criticism. This invites questions about what information, communicated by who and through which media, that is called for.

In discussions about vaccine hesitancy, concerns about misinformation spread *via* the internet are prevalent (30, 31). There is no doubt that decision-making processes about health-related issues (75, 76) including vaccination (27, 29, 61) today occur in a context where the internet, including social media, is commonly engaged. Information shared *via* social media, by various sources including public health communicators (77) and groups gathering vaccine hesitant actors (27) and private persons (28), may affect such decision-making processes, by influencing assessments of benefits and risks (26). The current study provides only a blunt measure of the importance of social media for HPV vaccination decision-making, as the survey inquired only about the perceived reliability of social media as a whole, while not differentiating between different actors, yielding any information about the actual seeking of or reliance on such information, nor accounting for social aspects of health-related communication online. This study does however affirm the presence of contestation of expert knowledge, expressed in the noted correlation between vaccine hesitancy and lack of trust in healthcare professionals, regulatory institutions and researchers, which has been observed in other studies to be interwoven with the reliance on information from social media (22, 24). In this context it is perhaps not self-evident that more information from healthcare providers will decrease hesitancy. In fact, this study found no conclusive difference between those expressing vaccine hesitancy or not in terms of whether they had received information from healthcare providers about any risks associated with the HPV vaccine, and vaccine non-hesitancy was associated with reporting having received no information at all about risks associated with the HPV vaccine, from healthcare providers or elsewhere. Other studies have found that providing additional information about the HPV vaccine including its potential side effects could cause vaccine hesitancy to decrease, remain unchanged, or increase (78, 79). It has also been noted that vaccine hesitancy and other forms of contemporary contestation of expert knowledge often entail an interest in and attachment to medical science rather than simple hostility to it (24, 36). Thus, while providing factual and transparent information about the HPV vaccine remains a priority (10, 28, 42), the issue of trust remains.

Regarding the evaluation of the trustworthiness of different sources of information about the HPV vaccine, vaccine hesitancy was associated with a higher rating of CAM sources. A link between vaccine hesitancy and CAM use has been affirmed elsewhere (80, 81). In this study, the association between vaccine hesitancy and lack of trust in medical professionals and regulatory institutions was much stronger, however. This is in correspondence with Hornsey et al.'s (81) identification of a stronger correlation between vaccine hesitancy and distrust in conventional medical treatments, than between such hesitancy and trust in CAM. Hornsey et al. (81) take this as an indication of a “push” into vaccine hesitancy, due to mistrust in conventional medicine, over and above a “pull” due to trust in CAM, and they therefore point to the importance of increasing our understanding of that mistrust.

Larson et al. (58) regard trust in vaccines as the result of a complex interaction not only between trust in the product, healthcare providers and medical policy-makers, but also a more general trust in societal institutions at large, in relation to which they emphasize the importance of past experiences. When these various aspects of trust do not align, trust in vaccinations is likely to weaken, which in turn may lead to trust instead being placed in other, including vaccine hesitant, sources or influences (58). This argument is clearly in line with the association between vaccine hesitancy and weaker levels of confidence in societal institutions found in this study. Along similar lines, Goldenberg (25) affirms not only that poor trust in medical and scientific institutions is fundamental to contemporary vaccine hesitancy, but also that this understanding offers a more constructive approach to the issue than dominant notions of a public knowledge deficit.

One of the contexts in which Goldenberg (25) places the current lack of trust in science and medicine is historical and contemporary structures of inequality and injustice. Correspondingly, higher levels of vaccine hesitancy among less privileged groups in society have been tied to lower levels of trust in healthcare institutions due to historical or personal experiences of being deprioritized or even exploited by official institutions, including healthcare (56, 57, 82). In Mohottige and Boulware's words (83), mistrust can be seen as a byproduct of inequitable systems. While the importance of such experiences of inequity within the Swedish context can be debated, it is worth reflecting on what might be the origins and implications of people feeling that their interests are not necessarily served or highly prioritized by healthcare and other societal institutions.

On the institutional level, a factor found to have affected vaccine confidence in Sweden (39, 42, 55) and elsewhere (21, 57) is concern with financial interests being a factor in healthcare research and practice. Such worries about profit being a motive in drug development and research are noted by Hausman (36) as she refers to vaccine hesitancy reflecting concerns that are not uncommon in the US population. Accordingly, Wheeloch and Ives (84) argue that efforts to increase vaccine confidence should include medical research and practice demonstrating trustworthiness through renewed measures to curb financial conflicts of interests in biomedical, including vaccine, research. Similar arguments about the relevance of increasing the transparency (37) and maintaining the trustworthiness of healthcare practice and research are made by Heneghan and McCartney (85) and Warren et al. (86) as well as Goldenberg (25, 32). Along related lines, in the sense of turning the gaze toward the “us” of healthcare institutions and practices rather than toward the vaccine hesitant “them” (25), Southwell et al. (30) argue that instead of focusing solely on policing problematic health-related online communication by actors external to conventional medicine, efforts should be aimed at addressing vulnerabilities and inadequacies in health information systems, including issues related to lack of trust in healthcare.

On the institutional and the individual level, apart from promoting trust by providing transparent and factual information about the HPV vaccine, including about potential side effects (10, 28, 42) or uncertainties (37), healthcare providers should avoid forms of interaction and communication that discourage confidence. This can include developing self-scrutiny (25) or self-reflexivity (83), awareness of medical mistrust (56) and of diverse and sometimes conflicting views on vaccines (10), and communication strategies which allow for listening to (83) and being willing to discuss vaccine concerns with patients (37, 42). While the attitudes of strongly vaccine hesitant people with very low confidence in societal institutions are presumably unlikely to be affected by such renewed communication strategies, those with milder hesitancy and a more ambivalent attitude toward healthcare and other institutions may. Conversely, failed communication with the latter group can exacerbate hesitancy (87). This is supported by studies showing that mothers who believed that their doctors would take their vaccine concerns seriously were more likely to accept the HPV vaccine (45), while vaccine hesitancy or refusal has been noted to arise due to feeling dismissed or disrespected by caregivers (88).

Overarchingly, Goldenberg (25, 32) emphasizes the importance of healthcare providers moving away from one-way didactic models of outreach, aiming at remedying vaccine hesitancy understood in terms of a knowledge deficit, in favor a more dialogical approach [also (33, 89)]. The latter can enable responsiveness to concerns of the individual or the public in order to support the relationship of trust that is necessary for public health interventions to work (90). Such an approach is not supported, however, by common and oppositional framings of the issue as a conflict or war between pro- and anti-vaxxers [also (36)] or between science and ignorance, as this yields limited space for mutual understanding or workable solutions, while likely hardening vaccine hesitant attitudes and mistrust. In addition to providing more space for alignment between public health agendas and public concerns, a less oppositional or polarizing stance can also, as Goldenberg (25, 32) argues, reaffirm and re-center the position of the scientific and medical expert in relation to the public.

### Limitations

While this study was distributed to a web panel consisting of randomly selected participants deemed to be nationally representative of those with regular access to internet, the response rate was limited at 37%. It is not unlikely that those with a negative, or for that matter a decisively positive, attitude toward HPV vaccination were more likely to participate. This is an issue common to most survey studies, however, and the response rate was similar to other studies conducted by Kantar Sifo. In addition, the survey included, and was introduced as also concerning, questions about another medical reproductive intervention, the copper IUD, which would somewhat decrease the risk of self-selection of women with a particular interest in HPV vaccination. The results were also weighted using national weights, pertaining to age and region, provided by Statistics Sweden.

This study provides a limited basis on which to draw conclusions about people's (future) decisions on whether or not to receive HPV vaccination. As vaccine hesitancy and vaccine refusal are not identical phenomena (25), we do not know if those reporting positive attitudes toward HPV vaccination will vaccinate their children in the future, and vice versa.

## Conclusion and Practice Implications

This survey study shows a positive attitude toward HPV vaccination in Sweden, alongside a presence of vaccine hesitancy associated with lower education and lower income and, particularly, a lower level of confidence in societal institutions including healthcare professionals, institutions regulating medical practice and researchers. Our results support the importance of providing transparent information about the HPV vaccination, and mitigating institutional- and individual-level practices in healthcare that do not promote trust. We underscore the importance of healthcare professionals being open to listen to and discuss vaccine concerns with patients.

## Data Availability Statement

The raw data supporting the conclusions of this article are available via the corresponding author, upon reasonable request.

## Ethics Statement

The study involved human participants and was reviewed and approved by Swedish Ethical Review Authority. Informed consent for participation was provided through the completion of the survey and submission of responses.

## Author Contributions

MW coordinated the design of the study, the analysis and interpretation of results, and the writing of the manuscript. LG contributed to the design of the study, to the interpretation of results, and to the revision of the manuscript. Both authors have approved the final version of the manuscript.

## Funding

This research was supported by FORTE—Swedish Research Council for Health, Working Life and Welfare (Dnr 2018-00951).

## Conflict of Interest

The authors declare that the research was conducted in the absence of any commercial or financial relationships that could be construed as a potential conflict of interest. The reviewer M-MH declared a shared affiliation with the authors, to the handling editor at the time of review.

## Publisher's Note

All claims expressed in this article are solely those of the authors and do not necessarily represent those of their affiliated organizations, or those of the publisher, the editors and the reviewers. Any product that may be evaluated in this article, or claim that may be made by its manufacturer, is not guaranteed or endorsed by the publisher.
